# Prevalence of Post-Stroke Cognitive Impairment in China: A Community-Based, Cross-Sectional Study

**DOI:** 10.1371/journal.pone.0122864

**Published:** 2015-04-13

**Authors:** Yanji Qu, Lin Zhuo, Na Li, Yiqing Hu, Weihua Chen, Yun Zhou, Jinwei Wang, Qingmei Tao, Jing Hu, Xiaolu Nie, Siyan Zhan

**Affiliations:** 1 Guangdong Cardiovascular Institute, Guangdong General Hospital, Guangdong Academy of Medical Sciences, Guangzhou, China; 2 Department of Epidemiology and Bio-Statistics, Peking University Health Science Center, Beijing, China; 3 Fangshan District Center for Disease Control and Prevention, Beijing, China; 4 Bansongyuan Center of Community Health Services, Huangpu District, Shanghai, China; 5 East Nanjing Road Center of Community Health Services, Huangpu District, Shanghai, China; 6 Department of Neurology, Beijing Tiantan Hospital, Capital Medical University, Beijing, China; Institute of Psychiatry, UNITED KINGDOM

## Abstract

International hospital-based studies have indicated a high risk of cognitive impairment after stroke, evidence from community-based studies in China is scarce. To determine the prevalence of post-stroke cognitive impairment (PSCI) and its subtypes in stroke survivors residing in selected rural and urban Chinese communities, we conducted a community-based, cross-sectional study in 599 patients accounting for 48% of all stroke survivors registered in the 4 communities, who had suffered confirmed strokes and had undergone cognitive assessments via the Montreal Cognitive Assessment (MoCA), Mini-Mental State Examination (MMSE), and Hachinski Ischemia Scale (HIS). Detection of PSCI was based on scores in these neuropsychological scales. Factors potentially impacting on occurrence of PSCI were explored by comparing demographic characteristics, stroke features, and cardiovascular risk factors between patients with and without PSCI. The overall prevalence of PSCI was 80.97% (95%CI: 77.82%-84.11%), while that of non-dementia PSCI (PSCI-ND) and post-stroke vascular dementia (PSD) was 48.91% (95%CI: 44.91%-52.92%) and 32.05% (95%CI: 28.32%-35.79%), respectively. Prior stroke and complications during the acute phase were independent risk factors for PSCI. The risk of recurrent stroke survivors having PSCI was 2.7 times higher than for first-episode survivors, and it was 3 times higher for those with complications during the acute phase than for those without. The higher prevalence of PSCI in this study compared with previous Chinese studies was possibly due to the combined effects of including rural stroke survivors, a longer period from stroke onset, and different assessment methods. There is an urgent need to recognize and prevent PSCI in stroke patients, especially those with recurrent stroke and complications during the acute phase.

## Introduction

Stroke is a major cause of disability and mortality for adults worldwide [1], and is the second most common cause of cognitive impairment, just behind neurological disease. Stroke has been reported to increase the risk of cognitive impairment at least 5 to 8 times [ 2 – 3 ]. Although the prevalence of post-stroke cognitive impairment (PSCI) has been studied in different countries [4–15], the results are far from consistent due to differences in patient characteristics, neuropsychological detection methods, sample sizes, and analytical methods. In recent decades, epidemiological studies have suggested an increase in the incidence of stroke in China [16], which may result in an increased prevalence of PSCI. Data on PSCI in China are scarce, especially at the community level. The aim of this community-based, cross-sectional, observational study was to determine the prevalence of PSCI and its subtypes in stroke survivors residing in selected rural and urban Chinese communities and explore the impact of potential risk factors.

## Materials and Methods

### Ethics statement

The study was approved by the Ethical Committee of Peking University Health Science Center and was performed in accordance with the ethical standards set out in the Declaration of Helsinki. Written informed consents were obtained from all included stroke survivors or their surrogates.

### Study areas

**Fig 1:**
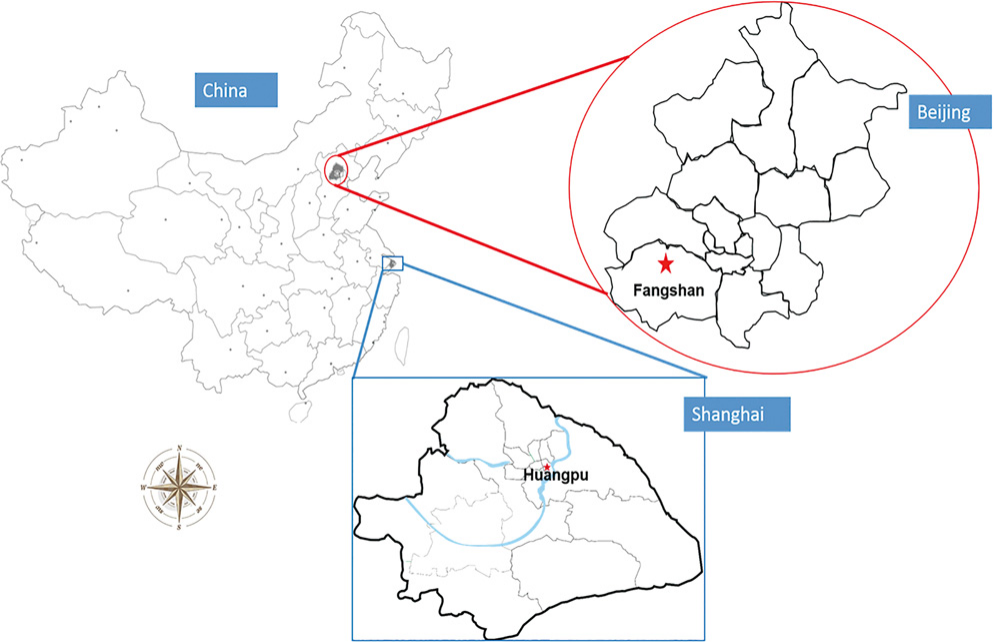
Location of the study area. In the 2 areas studied, stroke, hypertension, coronary artery disease, and diabetes are classified as community-managed chronic diseases [18–19]. This means patients will get higher reimbursement ratio in Community Health Service Centers (CHSCs) (90% to 100% in CHSCs VS 40% in other hospitals) and more convenience for referral to the senior hospitals. So, stroke survivors are obligated to register in CHSCs for primary rehabilitation care. Meanwhile, CHSCs are responsible to report patients with the four chronic diseases to the surveillance center, usually the Center for Disease Control (CDC). We used the registered information in CHSCs and an adequate number of cases was available from the CHSCs in the 2 selected areas.

Four communities, 2 located in the Fangshan District of Beijing and 2 in the Huangpu District of Shanghai were selected as the rural and urban areas for the study (Fig 1). Fangshan District (39°30'–39°55' N, 115°25'–116°15' E), which has an area of 2019 square kilometers and a population of 986,000, is situated 45 kilometers southwest of downtown Beijing. This region, 60% of which is mountainous, was considered representative of the rural northern part of China. As with the ‘stroke belt’ of southeastern USA, this region is located in the ‘stroke belt’ of China [17]. Huangpu District, which has an area of 20.5 square kilometers and a permanent resident population of 909,000, is located in downtown Shanghai and is an economic, administrative and cultural center. People residing in Huangpu District have a lifestyle typical of metropolitan residents, and the incidence of chronic non-infectious diseases in this population is high.

### Sample size estimation

According to the sample size calculation formula for cross-sectional studies:

$$N\;=\;\frac{Z_{a}^{2}\;\cdot \;pq}{d^{2}}$$

in which *p* is the estimated prevalence of PSCI, *q* = 1-*p*, *d* is the allowable error, and *Z*_*a*_ is the significance testing statistic, the sample size required for the study was determined to be 380 based on an estimation that the prevalence of PSCI was 38% [20] with a confidence interval (CI) of 95% and *d* = 0.05, a = 0.05 and *Z*_*a*_ = 1.96. We subsequently enlarged the sample size by a multiple of 1.5 to 570 taking account of the impact of non-strict cluster sampling.

### Diagnosis of PSCI

**Fig 2:**
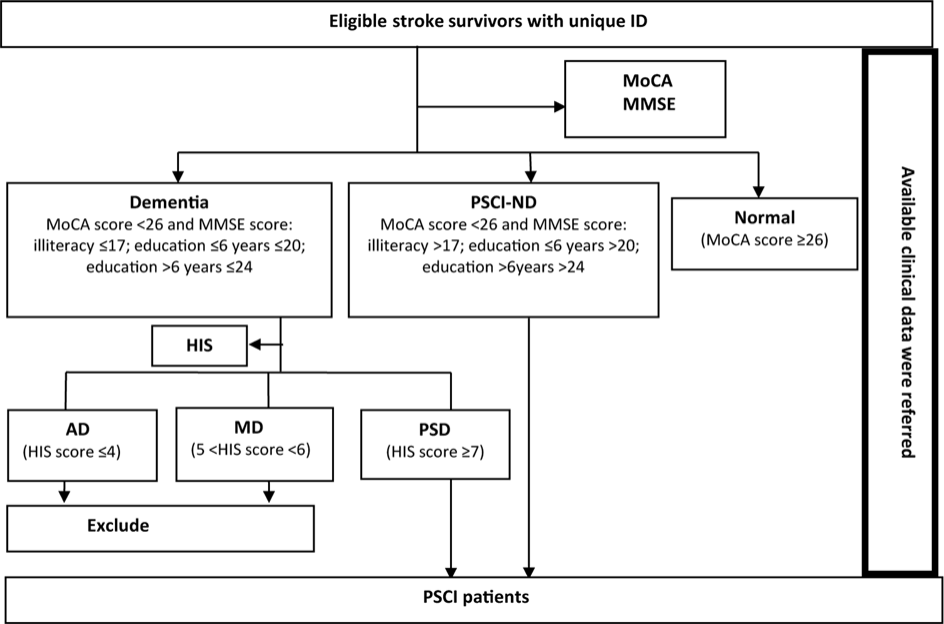
Flow chart illustrating the diagnosis of PSCI.

PSCI was diagnosed by a neurologist mainly on the basis of neuropsychological scale scores together with other available clinical information. The Montreal Cognitive Assessment (MoCA) score with its accepted cut-off of 26 was used to detect mild cognitive impairment (MCI). It was reported that the sensitivity and specificity of MoCA with the cut-off 26 was 90% and 87% respectively when used to screen MCI patients in Canada [21] and its good reliability and validity were also confirmed in China [22–24]. The Mini-Mental State Examination (MMSE) was used to distinguish non-dementia PSCI (PSCI-ND) and dementia among subjects with cognitive impairment. A meta-analysis which included 37 large sample dementia studies and 5 MCI studies in the recent 10 years found that the sensitivity and specificity of MMSE was 83.3% and 86.6% respectively when used to diagnose dementia patients based on community and primary hospitals [25]. Chinese Dementia and Cognitive Impairment Diagnosis and Treatment Guideline recommended MMSE for dementia screening (Grade A recommendation). The Cut–off stratified according to educational levels modified by Shanghai Mental Health Center in China was used [26]. Dementia was considered present when the MMSE score was ≤17 for illiterate subjects, ≤20 for patients with a primary school education background, and ≤24 for those with junior high school education. The Chinese version of Hachinski Ischemia Scale (HIS) revised by the Scale Collaborative Group (Fan B, et al.) in 1988 was used to differentiate vascular dementia (VD) from Alzheimer dementia (AD). The recognized cut-offs were adopted for the HIS score, i.e. ≤4 indicating AD, ≥7 indicating VD, and a score between the two indicating mixed dementia (MD). The sensitivity and specificity was reported to be 90.91% and 98.97% [27]. The only patients with post-stroke vascular dementia (PSD) included in the study were those with VD. A flow chart illustrating the diagnosis of PSCI in the eligible stroke survivors is shown in Fig 2.

### Participants eligibility and enrolment

Stroke survivors who had been referred to tertiary or secondary hospitals with a diagnosis of stroke confirmed by computerized tomography (CT) or magnetic resonance imaging (MRI), but not transient ischemic attacks (TIAs), and who were registered at CHSCs were potentially eligible for enrolment in the study. Patients with the following conditions were excluded: (1) known dementing illnesses history before stroke or existing neurological or psychiatric disorders; (2) comorbidities that potentially affect cognitive function, such as tumor-related strokes, hemopathological disorders, and serious liver or kidney dysfunction; (3) a history of alcohol or drug abuse and chemical (e.g. pesticide) poisoning; (4) communication problems hindering the performance of cognitive tests such as aphasia, apraxia, visual deprivation, hearing loss and unconsciousness, etc.; and (5) refusal to participate with the requirements of the study.

Eligible stroke survivors at the CHSCs were selected for cognitive assessment via telephone calls with general medical practitioners, and all were assigned a unique identification (ID) number. A structured questionnaire was completed by all participating patients with the help of their relatives to obtain information on demographic characteristics (age, gender, married status, education, family details, socioeconomic level, and insurance), cardiovascular disease risk factors (smoking, alcohol use, diet, exercise), stroke features [frequency of onset, type of stroke (ischemic, hemorrhagic or mixed), lesion location, complications during the acute phase (urinary incontinence, epilepsy, pseudo bulbar palsy, dyskinesia, depression)], and cerebrovascular disease risk factors (hypertension, hyperlipidaemia, coronary heart disease, atrial fibrillation, anaemia, diabetes, and arteriosclerosis). The questionnaires were followed by anthropometric measurements (height, weight, blood pressure) using standard methods. Subsequently, cognitive function was assessed by trained investigators or general practitioners in a relatively quiet room via use of the MMSE, MoCA and HIS scales. Limited neurological symptoms and signs in the HIS assessment were confirmed by a senior physician after physical examination.

### Disease and risk factor definitions

Stroke was defined according to the commonly used World Health Organization (WHO) criteria [28]. Overweight was defined as a body mass index (BMI) ≥24 kg/m^2^, while a history of smoking was defined as at least 1 cigarette per day for more than 6 months, smoking cessation as stopping smoking for more than 6 months, and occasional smoking as a cessation time less than 6 months. A similar definition was applied for alcohol use. In addition to routine physical exercises, walking for more than 40 minutes a day was also counted as exercise in rural communities lacking sports facilities. However, household duties and physical labor were not counted as exercise.

### Quality assurance

The study team comprised neurologists, epidemiologists, physicians, general practitioners, and students at Peking University. All personnel were trained and certified. Application of the questionnaire and the neuropsychological scales was undertaken under the supervision of neurologists. A pilot test in 46 stroke survivors was carried out (on 26 April, 2012) to verify the validity of the procedures. Every completed questionnaire was checked against the registration information from the CHSCs. Inconsistencies were resolved by further consultation with the stroke survivors and/or their family members. All data were double-entered via Epidata which permitted automated flagging of inconsistent or questionable values. Data inconsistencies were resolved by checking the original questionnaires and scales.

### Statistical analysis

Prevalence of PSCI was calculated using the number of PSCI patients as the numerator and number of participants as the denominator. Poisson distribution was adopted to estimate the 95% confidential interval (CI) of the prevalence. A *χ* ^*2*^ test or Fischer’s exact test was applied for categorical variables and Student’s *t*-test for continuous variables. One-way analysis of variance and multivariate logistic regression were used to explore risk factors for PSCI. A multilevel logistic regression model was used to identify the hierarchical differences of the prevalence of PSCI in communities and individual levels. All analyses were performed using SPSS^®^ 22.0 (IBM Co. Ltd).

## Results

### Patient characteristics

During the period from July 2012 to July 2013, 607 registered stroke survivors, 307 from rural communities and 300 from urban communities, were evaluated. However, 8 patients, 3 from rural areas and 5 from urban areas were excluded due to the presence of severe aphasia. Thus, a total of 599 patients who fulfilled the eligibility criteria were included in the study and underwent investigations and cognitive tests. These patients accounted for about 48% of all registered stroke survivors in the 4 communities. Reasons for not considering the remainder included moving away from the community, absence from home for a period, mobility limitations, and the non-availability of caregivers to provide support. When patients who were included in the study were compared with those who were not, we found no differences in their ages and gender. The demographic characteristics, stroke features, and related cardiovascular risk factors of the participating patients are shown in Table 1. Some significant differences between rural and urban patients were observed for certain demographic characteristics (age, ethnicity, BMI, education level, employment, marital status, housing conditions, and annual income), stroke features (time since last onset, number of lesions, and location of lesions), and cardiovascular risk factors (hypertension, arteriosclerosis, smoking history, alcohol use, diet, and exercise).

**Table 1:** Demographic characteristics, stroke features, and related cardiovascular risk factors of patients who participated in the study.

	Total (*n* = 599)	Rural (*n* = 304)	Urban (*n* = 295)	*χ* ^*2*^/*t*	*p-*value
**Demographic profile**					
Gender (female)	324 (54%)	153 (50%)	171 (58%)	3.52	0.07
Age(Year)	67.91±16.57	61.89±8.99	74.05±19.95	-9.56	0.00*
Ethnicity (Han)	589 (98%)	295 (97%)	294 (99.7%)	6.27	0.02*
BMI ≥24 kg/m^2^	344 (58%)	221 (73%)	123 (42%)	60.58	0.00*
Education					
Illiteracy	101 (16.9%)	58 (19.1%)	43 (14.6%)	15.35	0.00*
1–6 years	162 (27%)	99 (32.6%)	63 (21.4%)		
>6 years	336 (56.1%)	147 (48.4%)	189 (64.1%)		
Employment status (employed)	39 (6.5%)	27 (8.9%)	12 (4.1%)	5.70	0.02*
Marital status (spouse living)	439 (73.3%)	249 (81.9%)	190 (64.4%)	23.42	0.00*
Housing conditions (solitude)	65 (10.9%)	17 (5.6%)	48 (16.3%)	17.65	0.00*
Annual household income (Yuan)	40486.3±35476.5	18736.5±17673.4	62684.6±35352.4	-19.01	0.00*
Annual personal income (including endowment insurance, Yuan)	17638.8±17169.9	4710.4±5384.9	30921.5±14818.3	-28.45	0.00*
Medical insurance (self-pay)	5 (0.8%)	2 (0.7%)	3 (1.0%)	0.23	0.68
**Stroke features**					
Stroke frequency					
First-ever stroke	426 (71.2%)	213 (70.3%)	213 (72.2%)	0.27	0.65
Recurrent stroke	172 (28.8%)	90 (29.7%)	82 (27.8%)		
Time since last onset of stroke (Year)	4.5±4.3	6.4±4.8	2.6±3.2	8.92	0.00*
Type of stroke					
Hemorrhagic	62 (10.4%)	38 (12.5%)	24 (8.1%)	3.11	0.21
Ischemic	518 (86.5%)	257 (84.5%)	261 (88.5%)		
Mixed	19 (3.2%)	9 (3.0%)	10 (3.4%)		
Number of lesions (multiple)	170 (28.4%)	36 (11.8%)	134 (45.4%)	83.07	0.00*
Location of lesions					
Telencephalon	261 (43.6%)	43 (14.1%)	218 (73.9%)	13.17	0.00*
Brainstem	16 (2.7%)	7 (2.3%)	9 (3.1%)		
Cerebellum	59 (9.9%)	8 (2.6%)	51 (17.3%)		
Diencephalon	1 (0.2%)	1 (0.3%)	0		
Unclear	262 (43.7%)	245 (80.6%)	17 (5.8%)		
Complications during the acute phase	295 (49.2%)	158 (52.0%)	137 (46.4%)	1.83	0.19
**Cardiovascular risk factors**					
Hypertension	505 (84.3%)	268 (88.2%)	237 (80.3%)	6.92	0.01*
Hyperlipidemia	236 (39.4%)	125 (41.1%)	111 (37.6%)	0.76	0.38
Coronary heart disease	218 (36.4%)	113 (37.2%)	105 (35.6%)	0.16	0.69
Arrhythmia	39 (6.5%)	23 (7.6%)	16 (5.4%)	1.13	0.29
Diabetes	150 (25.0%)	82 (27.0%)	68 (23.1%)	1.23	0.27
Arteriosclerosis	209 (34.9%)	80 (26.3%)	129 (43.7%)	19.98	0.00*
Anemia	11 (1.8%)	6 (2.0%)	5 (1.7%)	0.07	0.80
Smoking history					
Never	368 (61.4%)	151 (49.7%)	217 (73.6%)	38.12	0.00*
Cessation	118 (19.7%)	80 (26.3%)	38 (12.9%)		
Occasional	18 (3.0%)	9 (3.0%)	9 (3.0%)		
Frequent	95 (15.9%)	64 (21.1%)	31 (10.5%)		
Alcohol use					
Never	422 (70.5%)	192 (63.2%)	230 (78.0%)	30.30	0.00*
Cessation	85 (14.2%)	59 (19.4%)	26 (8.8%)		
Occasional	59 (9.8%)	26 (8.6%)	33 (11.2%)		
Frequent	33 (5.5%)	27 (8.9%)	6 (2.0%)		
Diet type					
Vegetarian	272 (45.4%)	169 (55.6%)	103 (34.9%)	28.69	0.00*
Normal	237 (39.6%)	91 (29.9%)	146 (49.5%)		
Meatier	90 (15.0%)	44 (14.5%)	46 (15.6%)		
Diet flavor					
Light	215 (35.9%)	124 (40.8%)	91 (30.8%)	69.63	0.00*
Normal	194 (32.4%)	52 (17.1%)	142 (48.1%)		
Salty	190 (31.7%)	128 (42.1%)	62 (21.0%)		
Exercise					
Inactivity	263 (43.9%)	84 (27.6%)	179 (60.7%)	143.34	0.00*
Frequent	95 (15.9%)	26 (8.6%)	69 (23.4%)		
Every day	241 (40.2%)	194 (63.8%)	47 (15.9%)		

* Statistically significant difference between the rural and urban groups (*p* < 0.05).

### Prevalence of PSCI and its impact factors

A total of 485 stroke survivors were found to have cognitive impairment on the basis of the MoCA score. Of these, 293 had PSCI-ND and 192 had dementia on the basis of the MMSE scores. All patients with dementia had HIS scores ≥7 and were classified as having PSD. Thus, the overall prevalence of PSCI was 80.97% (95%CI: 77.82%–84.11%), and it was 48.91% (95%CI: 44.91%–52.92%) and 32.05% (95%CI: 28.32%–35.79%) for PSCI-ND and PSD respectively. The proportion of PSCI-ND and PSD in all PSCI was 60.41% and 39.59% separately. There were 284 and 201 PSCI patients in the rural and urban community, giving the prevalence of PSCI in rural and urban community of 93.42% (95%CI: 90.63%–96.21%) and 68.14% (95%CI: 62.82%–73.45%), respectively.

In one-way analyses of variance, significant associations were found between PSCI and age, BMI, education, annual income, stroke frequency, time since last onset, number of lesions, complications during the acute phase, hypertension, and exercise. Detailed results for these analyses are provided in Online Resource Supplemental Table 1. These variables and whether the patients resided in a rural or urban community were included in the multivariate logistic regression analysis. The results of this analysis indicated that only stroke frequency and stroke complications in the acute phase were significant factors impacting on PSCI. The prevalence of PSCI was higher in both recurrent stroke survivors (OR = 2.74; *p* = 0.002) and those with complications during the acute phase (OR = 3.05; *p* = 0.000). (Table 2)

**Table 2:** Results of multivariable analysis exploring the risk factors impacting on PSCI.

Potential Factors impacting on PSCI		*OR*	95%*CI* of *OR*	*P*-value
**Resident areas** (ref: Rural)	Urban	0.55	0.16–1.89	0.340
**Age, years** (ref: <65)	≥65	1.11	0.62–1.99	0.731
**BMI, kg/m** ^**2**^ (ref: <24)	≥24	1.14	0.68–1.91	0.624
**Education, years** (ref: ≤6)	>6	0.93	0.54–1.59	0.778
**Annual household income, Yuan** (ref: ≤10,000)	10,000–30,000	0.62	0.22–1.78	0.376
	>30,000	0.60	0.20–1.81	0.367
**Annual personal income, Yuan** (ref: ≤10,000)	10,000–30,000	0.50	0.14–1.76	0.278
	>30,000	0.31	0.08–1.20	0.089
**Stroke frequency** (ref: First-ever stroke)	Recurrent stroke	2.74	1.47–5.11	0.002*
**Time since stroke onset** (ref: ≤3 months)	3–6 months	0.54	0.19–1.54	0.248
	6–12 months	0.72	0.31–1.66	0.439
	1–3 years	1.21	0.54–2.69	0.640
	>3 years	1.87	0.80–4.39	0.148
**Number of stroke lesions** (ref: Focal)	Multiple	0.66	0.38–1.12	0.124
**Complications in acute phase** (ref: Negative)	Positive	3.05	1.84–5.05	.000*
**Hypertension** (ref: Negative)	Positive	1.19	0.64–2.20	0.590
**Exercise** (ref: Inactivity)	Frequent	0.84	0.45–1.57	0.590
	Every day	0.83	0.44–1.56	0.554

* Statistically significant; ref: reference.

In view of the hierarchical structure of the data, we also constructed a multilevel logistic regression model to determine whether there was variance of PSCI at a community level. However, we found no significant difference between the selected rural and urban communities (Z = 0.70, *p* = 0.486).

## Discussion

The overall prevalence of PSCI was 80.97% in this study, while that of PSCI-ND and PSD was 48.91% and 32.05%, respectively. In comparison, the prevalence of PSCI in previously reported studies in various countries has varied from 17% to 92% [4–15, 29]. This wide variation can be attributed mainly to differences in study areas, time since stroke onset, stroke type, assessment methods, and diagnostic criteria (Table 3). In other studies of PSCI in China, hospital-based cohort studies by Zhou et al. [ 13 ] and Zhang et al. [ 14 ] found that the incidence of PSCI at 3 months after stroke was 32.2% and 27.9%, respectively, while in an urban community-based, cross-sectional study, Tu et al. [15] reported that the prevalence of PSCI in ischemic stroke survivors aged ≥40 years was 41.8%. The prevalence of PSCI in our study was consistent with the range of previously reported values, but was higher than rates reported in previous Chinese studies. There are several likely reasons for this, including firstly, different study areas. Unlike previous studies, we included stroke survivors from rural communities who, in comparison with patients residing in urban communities, usually responded with difficulty during cognitive tests. This was reflected in longer response times and unfamiliarity with the content of the tests. Although there was no significant difference in the prevalence of PSCI between the rural and urban communities based on our multilevel logistic regression model, the raw prevalence was higher in the rural communities (93.4% vs 68.1%). Secondly, the methods used to assess cognitive function were different. Several previous studies used MMSE criteria of ≤17 for illiteracy, ≤20 for primary school education, ≤22 for middle school education, and ≤23 for junior college and higher levels of education to detect PSCI. In the present study, an MoCA score of 26 was the threshold used, and MMSE scores of ≤17 for illiteracy, ≤20 for primary school education, and ≤24 for middle school and higher education were applied, while a HIS score of ≥7 was adopted to detect PSD. A recent population-based study that compared the MoCA and MMSE [ 30 ] found that MoCA could detect more vascular cognitive impairment in patients with TIAs and stroke than the MMSE, and MoCA is currently recommended over MMSE by the US National Institute of Neurological Disorder in the chronic post-stroke setting [ 31 ]. If MMSE criteria of ≤17 for illiteracy, ≤20 for primary school education, ≤22 for middle school education, and ≤23 for junior college and higher education had been used to detect PSCI in our study, the prevalence would have been 32.7%.

**Table 3:** Principal published studies of the prevalence of PSCI.

Study	Country	Study type	Patients	Cognitive assessment methods	Prevalence of PSCI	Risk factors
Madureira et al. (2001) [4]	Portugal	Hospital-based cohort study	Ischemic or hemorrhagic stroke survivors	MMSE, HDRS, BDS	55% at 3 months post-stroke	Older, lower educational level, more sided lesions
Tham et al. (2002) [5]	Singapore	Hospital-based cohort study	TIA or non-disabling ischemic stroke	VDB	44% at 6 months post-stroke	-
Patel et al. (2003) [6]	UK	Population-based observational study	First-ever stoke survivors	MMSE<24	39% at 3 months and 35%,30%,32% at 1, 2, 3 years	-
Sachdev et al. (2006) [7]	Australia	Case-control study	Stroke survivors aged 50–85 years	Detailed neuropsychological and medical psychiatric assessments	36.7% at 3 to 6 months post-stroke	-
Ihle-Hansen et al. (2011) [8]	Norway	Hospital-based cohort study	First-ever stroke and TIA survivors free from pre-stroke cognitive decline	-	57% at 1 year post-stroke	-
Mukhopadhyay et al. (2012) [9]	India	Community-based, cross-sectional study	Stroke survivors aged ≥60 years	MMSE <24	66.66%	-
Wong et al. (2012) [10]	Hong Kong	Prospective observational study	Aneurysmal subarachnoid haemorrhage	MoCA <26, MMSE	73% at 3 months post-stroke	-
Garcia et al. (2013) [11]	France	Hospital-based cross-sectional study	Spontaneous intracerebral haemorrhage	Informant Questionnaire on Cognitive Decline in the Elderly, Instrumental Activities of Daily Living, comprehensive clinical and neuropsychological assessment	70.51% at mean time since stroke of 40 months	Rankin score >1 at discharge, haemorrhage volume
Jacquin et al. (2014) [12]	France	Hospital-based prospective cohort study	Stroke patients without pre-stroke dementia, mild cognitive disorders, or severe aphasia	MMSE ≤26, MoCA ≤26, neuropsychological battery confirmed PSCI	47.3% at 3 months post-stroke	Age, low education level, a history of diabetes mellitus, acute confusion, silent infarcts, and functional handicap at discharge, MMSE and MOCA scores during hospitalization
Zhou et al. (2005) [13]	Chongqing, China	Hospital-based cohort study	Ischemic stroke survivors aged ≥55 years	Informant Questionnaire on Cognitive Decline in the Elderly and MMSE	32.2% at 3 months post-stroke (128/434)	Age, low educational level, prior stroke, every day drinking, dysphasia, left carotid territory infarction
Zhang et al. (2012) [14]	Multiple areas, China	Hospital-based cohort study	First-ever stroke survivors aged ≥45 years	Neuropsychological tests	27.49% at 3 months post-stroke	Older age, low educational level, depressive symptoms, obesity, stroke severity at 3 months post-stroke, and cortex lesions
Tu et al. (2011) [15]	Changsha, China	Community-based, cross-sectional study	Ischemic stroke survivors aged ≥40 years	MoCA, MMSE	41.8%	Age, low educational level, every day drinking, urinary incontinence, dyskinesia, not reading

BDS, Blessed Dementia Scale; HDRS, Hamilton Depression Rating Scale; MMSE, Mini-Mental State Examination; MoCA, Montreal Cognitive Assessment; TIA, transient ischemic attack; PSCI, post-stroke cognitive impairment; VDB, Vascular Dementia Battery;-, No reported.

Another possible reason for the higher PSCI prevalence in the present study is the longer time since stroke onset. The average time from onset of the last stroke in the patients we studied was 4.5 years, and in 39.5% it was more than 3 years. Previous studies have mainly focused on the prevalence of PSCI at 3 months post-stroke. However, while a high incidence of PSCI is commonly seen clinically at 3 months after stroke, this is often followed by improvement at 3 to 6 months but thereafter subsequent deterioration with the passage of time.

Several studies [ 4 , 11 – 15 , 32 – 34 ] have assessed risk factors that influence PSCI. Demographic characteristics (advanced age, female gender, lower education, poor economic conditions), cardiovascular disease risk factors (hypertension, hyperlipidaemia, coronary heart disease, atrial fibrillation), lifestyle and behavioural factors (alcohol use), stroke features (multiple lesions, recurrent stroke, and complications such urinary incontinence, dyskinesia, pseudo bulbar palsy, depression, etc.) have all been reported to be independent risk factors for PSCI. However, one study [ 35 ] found that PSCI was unrelated to vascular risk factors such as hypertension, diabetes, hyperlipidaemia, TIA, ischemic heart disease, and atrial fibrillation. We collected information on potential risk factors via a questionnaire and compared PSCI and non-PSCI stroke survivors. Consistent with previous studies [36–40], we found that prior stroke was a risk factor for PSCI, and that complications during the acute phase impacted independently on PSCI. The risk of PSCI in recurrent stroke survivors was 2.7 times that of first-episode patients, and in patients with complications during the acute phase, it was 3 times that of those without. Recurrence of stroke is usually accompanied by an extension of the lesion or deterioration of the lesion grade and a cognitive domain may be involved. In addition, complications during the acute phase were an important indicator of stroke severity which was related to PSCI. Thus, our study suggested that stroke characteristics and complications of the stroke were the main factors that influenced PSCI, whereas demographic characteristics and cardiovascular risk factors were not. This is consistent with the results of a meta-analysis [41].

This study had certain limitations. Firstly, as non-strict cluster sampling was adopted and not all stroke survivors registered in the 4 communities were involved in the study, there may have been some sampling bias. Meanwhile, we did not restrict research subjects to stroke survivors with unified stroke characteristics, such as stroke types, times of onset, time frame between stroke occurrence and cognitive testing. This would not hinder us from getting an average prevalence of PSCI in communities. Secondly, the participation rate of this study was low at 48% mainly due to moving away or absence for a while from the community and mobility limitations with no caregivers to provide support. Although no statistical significant was found when patients who were included in the study were compared with those who were not in their ages and gender, estimation bias could still exist. Thirdly, the stroke data was obtained from CHSCs in the study areas, and some important stroke features such as the location of lesions were not always available. Due to a lack of feasibility and limited availability of specialist equipment, we did not perform CT or MRI imaging on stroke survivors to confirm the diagnosis. Finally, although we had estimated the sample size in advance, the risk factor analysis was not considered and the sample size of the subgroups was relatively small, which may have affected the results. In the final analysis, this was a cross-sectional study that was relatively weak in exploring the potential impact of risk factors, and further community-based, longitudinal, prospective studies will be required to identify factors that are the most accurate predictors of PSCI. Prevalence was an index that was determined by the incidence and intervening mortality of the disease. As a cross-sectional study, intervening mortality of the stroke survivors was not available and we failed to consider the survival effects on the prevalence.

## Conclusions

The prevalence of PSCI in this study was higher than rates reported in previous Chinese studies, possibly due to the combined effects of including rural stroke survivors, and the use of different assessment methods. There is an urgent need to recognize and prevent PSCI in stroke patients, especially those with recurrent stroke and those with complications during the acute phase. As early preventive and treatment measures are expected to decrease the incidence of PSCI and delay the transition process of PSCI-ND to PSD, routine screening using simple standardized measures (e.g. the MMSE) is recommended in current stroke guidelines [42].

## Supporting Information

S1 Table Comparison of demographic characteristics, stroke features, and related risk factors between PSCI and non-PSCI patients. (DOC)

S2 Table Comparison of the difference between stroke survivors included and not in age and gender (DOC)

## References

[1] Strong K, Mathers C, Bonita R. Preventing stroke: saving lives around the world. Lancet Neurol. 2007; 6: 182–187. 17239805 10.1016/S1474-4422(07)70031-5

[2] Merino JG. Dementia after stoke: high incidence and intriguing associations. Stroke. 2002; 33: 2261–2262. 12219751

[3] Srikanth VK, Thrift AG, Saling MM, Anderson JF, Dewey HM, Macdonell RA, et al Increased risk of cognitive impairment 3 months after mild to moderate first-ever stroke: a community-based prospective study of nonaphasic English-speaking survivors. Stroke. 2003; 34: 1136–1143. 12702832 10.1161/01.STR.0000069161.35736.39

[4] Madureira S, Guerreiro M, Ferro JM. Dementia and cognitive impairment three months after stroke. Eur J Neurol. 2001; 8: 621–627. 11784347 10.1046/j.1468-1331.2001.00332.x

[5] Tham W, Auchus AP, Thong M, Goh ML, Chang HM, Wong MC, et al Progression of cognitive impairment after stroke: one year results from a longitudinal study of Singaporean stroke patients. J Neurol Sci. 2002; 203: 49–52. 12417356 10.1016/s0022-510x(02)00260-5

[6] Patel M, Coshall C, Rudd AG, Wolfe CD. Natural history of cognitive impairment after stroke and factors associated with its recovery. Clin Rehabil. 2003; 17: 158–166. 12625656 10.1191/0269215503cr596oa

[7] Sachdev PS, Brodaty H, Valenzuela MJ, Lorentz L, Looi JC, Berman K, et al Clinical determinants of dementia and mild cognitive impairment following ischaemic stroke: the Sydney Stroke Study. Dement Geriatr Cogn Disord. 2006; 21: 275–283. 16484805 10.1159/000091434

[8] Ihle-Hansen H, Thommessen B, Wyller TB, Engedal K, Øksengård AR, Stenset V, et al Incidence and subtypes of MCI and dementia 1 year after first-ever stroke in patients without pre-existing cognitive impairment. Dement Geriatr Cogn Disord. 2011; 32: 401–407. 10.1159/000335361 22311341

[9] Mukhopadhyay A, Sundar U, Adwani S, Pandit D. Prevalence of stroke and post-stroke cognitive impairment in the elderly in Dharavi, Mumbai. J Assoc Physicians India. 2012; 60: 29–32. 23777022

[10] Wong GK, Lam S, Ngai K, Wong A, Mok V, Poon WS, et al Evaluation of cognitive impairment by the Montreal cognitive assessment in patients with aneurysmal subarachnoid haemorrhage: prevalence, risk factors and correlations with 3 month outcomes. J Neurol Neurosurg Psychiatry. 2012; 83: 1112–1117. 10.1136/jnnp-2012-302217 22851612

[11] Garcia PY, Roussel M, Bugnicourt JM, Lamy C, Canaple S, Peltier J, et al Cognitive impairment and dementia after intracerebral hemorrhage: a cross-sectional study of a hospital-based series. J Stroke Cerebrovasc Dis. 2013; 22: 80–86. 10.1016/j.jstrokecerebrovasdis.2011.06.013 22421024

[12] Jacquin A, Binquet C, Rouaud O, Graule-Petot A, Daubail B, Osseby GV, et al Post-stroke cognitive impairment: high prevalence and determining factors in a cohort of mild stroke. J Alzheimers Dis. 2014; 40: 1029–1038. 10.3233/JAD-131580 24577459

[13] Zhou DH, Wang JY, Li J, Deng J, Gao C, Chen M. Frequency and risk factors of vascular cognitive impairment three months after ischemic stroke in China: the Chongqing Stroke Study. Neuroepidemiology. 2005; 24: 87–95. 15459515 10.1159/000081055

[14] Zhang Y, Zhang Z, Yang B, Li Y, Zhang Q, Qu Q, et al Incidence and risk factors of cognitive impairment 3 months after first-ever stroke: a cross-sectional study of 5 geographic areas of China. J Huazhong Univ Sci Technolog Med Sci. 2012; 32: 906–911. 10.1007/s11596-012-1056-9 23271295

[15] Tu QY, Yang X, Ding BR, Jin H, Lei ZH, Bai S, et al Epidemiological investigation of vascular cognitive impairment post ischemic stroke. Chinese Journal of Gerontology. 2011; 31: 3576–3579.

[16] Liu M, Wu B, Wang WZ, Lee LM, Zhang SH, Kong LZ. Stroke in China: epidemiology, prevention, and management strategies. Lancet Neurol. 2007; 6: 456–464. 17434100 10.1016/S1474-4422(07)70004-2

[17] Tang X, Hu Y, Chen D, Zhan S, Zhang Z, Dou H. The Fangshan/Family-based ischemic stroke study in China (FISSIC) protocol. BMC Med Genet. 2007; 8: 60 17825112 10.1186/1471-2350-8-60 PMC1997110

[18] Wang HB. Efficacy of free distribution of medicines for four chronic diseases for five years in a rural community in Beijing and suggestions for continuation. Chinese General Practice. 2014; 17(36): 4374–4377.

[19] Zhou JJ. Primary discussion of the comprehensive prevention and treatment pattern of the chronic non-communicable disease in Huangpu district, Shanghai. Shanghai Journal of Preventive Medicine. 2011; 23(5): 224–226.

[20] Qu YJ, Zhuo L, Zhan SY. Epidemiology characteristics of post-stroke cognitive impairment in China: a systematic review. Chin J Geriatr Heart Brain Vessel Dis. 2013; 15: 1294–1301.

[21] Nasreddine ZS, Phillips NA, Bédirian V, Charbonneau S, Whitehead V, Collin L, et al The Montreal Cognitive Assessment, MoCA: a brief screening tool for mild cognitive impairment. J Am Geriatr Soc. 2005; 53(4): 695–699. 15817019 10.1111/j.1532-5415.2005.53221.x

[22] Wen HB, Zhang ZX, Niu FS, Li L. The application of Montreal cognitive assessment in urban Chinese residents of Beijing. Chin J Intern Med. 2008; 47(1): 36–39. 18346324

[23] Jia GW, Song Q, Yin Y, Zhao HX, Wang L, Zhao RG, et al A preliminary study of application of Montreal cognitive assessment in Chongqing city. Neural Injury and Functional Reconstruction. 2008; 3 (1): 41–42.

[24] Zhang LX, Liu XQ. Determination of the cut-off point of the Chinese version of the Montreal cognitive assessment among Chinese elderly in Guangzhou. Chinese Mental Health Journal. 2008; 22 (2): 123–125.

[25] Mitchell AJ. A meta-analysis of the accuracy of the mini-mental state examination in the detection of dementia and mild cognitive impairment. J Psychiatr Res. 2009; 43: 411–431. 10.1016/j.jpsychires.2008.04.014 18579155

[26] Zhang M, Katzman R, Liu W. The prevalence of dementia and Alzheimer’s disease in Shanghai China: impact of age, gender and education. Ann Neurol. 1990; 27: 428–437. 2353798 10.1002/ana.410270412

[27] Fan B, Zhang MY, Wang ZY, Yao CD, Chi YF, Xu P. The application of Hachinski Ischemic Score in differentiation Alzheimer dementia and vascular dementia. Shanghai Archives of Psychiatry. 1989; 3: 131–135.

[28] Wu Z, Yao C, Zhao D, Wu G, Wang W, Liu J, et al Sino-MONICA project: a collaborative study on trends and determinants in cardiovascular diseases in China, Part i: morbidity and mortality monitoring. Circulation. 2001; 103: 462–468 11157701 10.1161/01.cir.103.3.462

[29] Pasi M, Poggesi A, Salvadori E, Pantoni L. Post-stroke dementia and cognitive impairment. Front Neurol Neurosci. 2012; 30: 65–69. 10.1159/000333412 22377866

[30] Pendlebury ST, Cuthbertson FC, Welch SJ, Mehta Z, Rothwell PM. Underestimation of cognitive impairment by Mini-Mental State Examination versus the Montreal Cognitive Assessment in patients with transient ischemic attack and stroke: a population-based study. Stroke. 2010; 41: 1290–1293. 10.1161/STROKEAHA.110.579888 20378863

[31] Wong GK, Lam S, Ngai K, Wong A, Mok V, Poon WS, et al Evaluation of cognitive impairment by the Montreal Cognitive Assessment in patients with aneurysmal subarachnoid haemorrhage: prevalence, risk factors and correlations with 3 month outcomes. J Neurol Neurosurg Psychiatry. 2012; 83: 1112–1117. 10.1136/jnnp-2012-302217 22851612

[32] Liu HJ, Fang XH, Qin XM, Mu LY, Zhang XQ, Li ST, et al Cognitive impairment and risk factor survey in patients with ischemic stroke in Beijing communities. Chinese Journal of Cerebrovascular Diseases. 2009; 6: 514–518.

[33] Xu Q, Lin Y, Geng JL, Li HW, Chen Y, Li YS. The prevalence and risk factors for cognitive impairment following ischemic stroke. Chinese Journal of Internal Medicine. 2008; 47: 981–984. 19134298

[34] Li JC, Zhou HD, Wang YJ. Impact of post-stroke dementia on the survival rate of the patients. Chinese Journal of Clinical Rehabilitation. 2005; 9: 156–158.

[35] Barba R, Martínez-Espinosa S, Rodríguez-García E, Pondal M, Vivancos J, Del Ser T. Poststroke dementia: clinical features and risk factors. Stroke. 2000; 31: 1494–1501. 10884443 10.1161/01.str.31.7.1494

[36] Pohjasvaara T, Erkinjuntti T, Ylikoski R, Hietanen M, Vataja R, Kaste M. Clinical determinants of poststroke dementia. Stroke. 1998; 29: 75–81. 9445332 10.1161/01.str.29.1.75

[37] Desmond DW, Moroney JT, Paik MC, Sano M, Mohr JP, Aboumatar S, et al Frequency and clinical determinants of dementia after ischemic stroke. Neurology. 2000; 54: 1124–1131. 10720286 10.1212/wnl.54.5.1124

[38] Tatemichi TK, Foulkes MA, Mohr JP, Hewitt JR, Hier DB, Price TR, et al Dementia in stroke survivors in the Stroke Data Bank cohort. Prevalence, incidence, risk factors, and computed tomographic findings. Stroke. 1990; 21: 858–866. 2349588 10.1161/01.str.21.6.858

[39] Kokmen E, Whisnant JP, O’Fallon WM, Chu CP, Beard CM. Dementia after ischemic stroke: a population-based study in Rochester, Minnesota (1960–1984). Neurology. 1996; 46: 154–159. 8559366 10.1212/wnl.46.1.154

[40] Desmond DW, Moroney JT, Sano M, Stern Y. Incidence of dementia after ischemic stroke. Results of a longitudinal study. Stroke. 2002; 33: 2254–2262. 12215596 10.1161/01.str.0000028235.91778.95

[41] Pendlebury ST, Rothwell PM. Prevalence, incidence, and factors associated with pre-stroke and post-stroke dementia: a systematic review and meta-analysis. Lancet Neurol. 2009; 8: 1006–1018. 10.1016/S1474-4422(09)70236-4 19782001

[42] Intercollegiate Stroke Working Party. National clinical guideline for stroke, 3rd edition. Royal College of Physicians.

