# Supplementary material for: Prevalence of Post-Stroke Cognitive Impairment in China: A Community-Based, Cross-Sectional Study
Source: PLoS One. 2015 Apr 13;10(4):e0122864. doi: 10.1371/journal.pone.0122864 (PMC4395303; doi:10.1371/journal.pone.0122864)
Supplement: S1 Table — (DOC) [file pone.0122864.s001.doc]

| **Table S1**. Comparison of demographic characteristics, stroke features, and related risk factors between PSCI and non-PSCI patients | | | | | | | | | | | | | | | | |
| --- | --- | --- | --- | --- | --- | --- | --- | --- | --- | --- | --- | --- | --- | --- | --- | --- |
|  | **Total (*n* = 599)** | | | |  | | **Rural (*n*= 304)** | | | |  | | **Urban (*n*= 295)** | | | |
|  | **PSCI**  **(*n* = 485)** | **N-PSCI**  **(*n* = 114)** | ***OR* (95% *CI*)** | ***p-*value** | | **PSCI**  **(*n* = 284)** | | **N-PSCI**  **(*n* = 20)** | ***OR* (95% *CI*)** | ***p-*value** | | **PSCI**  **(*n* = 201)** | | **N-PSCI**  **(*n* = 94)** | ***OR* (95% *CI*)** | ***p-*value** |
| **1. Demographic Characteristics** | | |  |  | |  | |  |  |  | |  | |  |  |  |
| **Gender:** |  |  |  |  | |  | |  |  |  | |  | |  |  |  |
| Male | 227 | 48 | 1.00 |  | | 140 | | 11 | 1.00 |  | | 87 | | 37 | 1.00 |  |
| Female | 258 | 66 | 0.83 (0.55-1.25) | 0.83 | | 144 | | 9 | 1.26 (0.51-3.13) | 0.62 | | 114 | | 57 | 0.85 (0.52-1.40) | 0.53 |
| **Age, years:** |  |  |  |  | |  | |  |  |  | |  | |  |  |  |
| <65 | 229 | 38 | 1.00 |  | | 182 | | 13 | 1.00 |  | | 47 | | 25 | 1.00 |  |
| ≥65 | 256 | 76 | 0.56 (0.36-0.86) | 0.01* | | 102 | | 7 | 1.04 (0.40-2.69) | 0.93 | | 154 | | 69 | 1.19 (0.68-2.08) | 0.55 |
| **Ethnicity:** |  |  |  |  | |  | |  |  |  | |  | |  |  |  |
| Han | 477 | 112 | 1.00 |  | | 277 | | 18 | 1.00 |  | | 200 | | 94 |  |  |
| Other | 8 | 2 | 0.94 (0.19-4.48) | 0.94 | | 7 | | 2 | 0.23 (0.04-1.18) | 0.08 | | 1 | | 0 | - | - |
| **BMI, kg/m^2^:** |  |  |  |  | |  | |  |  |  | |  | |  |  |  |
| <24 | 191 | 62 | 1.00 |  | | 75 | | 6 | 1.00 |  | | 116 | | 56 | 1.00 |  |
| ≥24 | 293 | 51 | 1.87 (1.23-2.82) | 0.00* | | 208 | | 13 | 1.28 (0.47-3.49) | 0.63 | | 85 | | 38 | 1.08 (0.66-1.78) | 0.76 |
| **Education, years:** |  |  |  |  | |  | |  |  |  | |  | |  |  |  |
| ≤6 | 226 | 37 | 1.00 |  | | 148 | | 9 | 1.00 |  | | 78 | | 28 | 1.00 |  |
| >6 | 259 | 77 | 0.55 (0.36-0.85) | 0.01* | | 136 | | 11 | 0.75 (0.30-1.87) | 0.54 | | 123 | | 66 | 0.67 (0.40-1.13) | 0.13 |
| **Employment status:** |  |  |  |  | |  | |  |  |  | |  | |  |  |  |
| Employed | 29 | 10 | 1.00 |  | | 23 | | 4 | 1.00 |  | | 6 | | 6 | 1.00 |  |
| Not employed | 456 | 104 | 1.51 (0.71-3.20) | 0.28 | | 261 | | 16 | 2.84 (0.88-9.19) | 0.08 | | 195 | | 88 | 2.22 (0.70-7.06) | 0.18 |
| **Marital status:** |  |  |  |  | |  | |  |  |  | |  | |  |  |  |
| Spouse living | 360 | 79 | 1.00 |  | | 235 | | 14 | 1.00 |  | | 125 | | 65 | 1.00 |  |
| Spouse not living | 125 | 35 | 0.78 (0.50-1.23) | 0.29 | | 49 | | 6 | 0.49 (0.18-1.33) | 0.16 | | 76 | | 29 | 1.36 (0.81-2.30) | 0.25 |
| **Housing conditions:** |  |  |  |  | |  | |  |  |  | |  | |  |  |  |
| Solitude | 47 | 18 | 1.00 |  | | 15 | | 2 | 1.00 |  | | 32 | | 16 | 1.00 |  |
| With family | 438 | 96 | 1.75 (0.97-3.14) | 0.06 | | 269 | | 18 | 1.99 (0.42-9.39) | 0.38 | | 169 | | 78 | 1.08 (0.56-2.09) | 0.81 |
| **Annual household income, Yuan:** | |  |  |  | |  | |  |  |  | |  | |  |  |  |
| ≤10000 | 152 | 9 | 1.00 |  | | 146 | | 8 | 1.00 |  | | 6 | | 1 | 1.00 |  |
| 10000-30000 | 116 | 19 | 0.36 (0.16-0.83) | 0.02* | | 82 | | 8 | 0.56 (0.20-1.55) | 0.27 | | 34 | | 11 | 0.52 (0.06-4.76) | 0.56 |
| >30000 | 215 | 84 | 0.15 (0.07-0.31) | 0.00* | | 56 | | 4 | 0.77 (0.22-2.65) | 0.68 | | 159 | | 80 | 0.33 (0.04-2.80) | 0.31 |
| **Annual personal income, Yuan** (including endowment insurance): | |  |  |  | |  | |  |  |  | |  | |  |  |  |
| ≤10000 | 267 | 18 | 1.00 |  | | 260 | | 17 | 1.00 |  | | 7 | | 1 | 1.00 |  |
| 1000-30000 | 157 | 55 | 0.19 (0.11-0.34) | 0.00* | | 22 | | 3 | 0.48 (0.13-1.76) | 0.27 | | 135 | | 52 | 0.37 (0.05-3.09) | 0.36 |
| >30000 | 59 | 40 | 0.10 (0.05-0.19) | 0.00* | | 2 | | 0 | - | 0.99 | | 57 | | 40 | 0.20 (0.02-1.72) | 0.14 |
| **Medical insurance:** |  |  |  |  | |  | |  |  |  | |  | |  |  |  |
| Self-pay | 5 | 0 |  |  | | 2 | | 0 |  |  | | 3 | | 0 |  |  |
| Insurance | 480 | 114 | - | - | | 282 | | 20 | - | - | | 198 | | 94 | - | - |
| **2. Stroke Features** |  |  |  |  | |  | |  |  |  | |  | |  |  |  |
| **Stroke frequency:** |  |  |  |  | |  | |  |  |  | |  | |  |  |  |
| First-ever stroke | 330 | 96 | 1.00 |  | | 193 | | 20 |  |  | | 137 | | 76 | 1.00 |  |
| Recurrent stroke | 154 | 18 | 2.49 (1.45-4.27) | 0.00* | | 90 | | 0 | - | - | | 64 | | 18 | 1.97 (1.09-3.57) | 0.03* |
| **Time since stroke onset:** | |  |  |  | |  | |  |  |  | |  | |  |  |  |
| ≤3 months | 37 | 17 | 1.00 |  | | 9 | | 2 | 1.00 |  | | 28 | | 15 | 1.00 |  |
| 3-6 months | 20 | 12 | 0.80 (0.28-2.35) | 0.57 | | 8 | | 0 | - | - | | 12 | | 12 | 0.51 (0.16-1.66) | 0.27 |
| 6-12 months | 66 | 26 | 1.44 (0.59-3.48) | 0.68 | | 26 | | 2 | 5.0 (0.42-59.66) | 0.20 | | 40 | | 24 | 1.12 (0.43-2.92) | 0.82 |
| 1-3 years | 148 | 31 | 2.74 (1.21-6.22) | 0.03* | | 70 | | 7 | 5.86 (0.71-48.68) | 0.10 | | 78 | | 24 | 2.04 (0.82-5.08) | 0.13 |
| >3 years | 206 | 27 | 4.73 (2.12-10.58) | 0.00* | | 163 | | 8 | 16.75 (2.08-134.85) | 0.01* | | 43 | | 19 | 1.29 (0.51-3.30) | 0.59 |
| **Type of stroke:** |  |  |  |  | |  | |  |  |  | |  | |  |  |  |
| Hemorrhagic | 54 | 8 | 1.00 |  | | 35 | | 3 | 1.00 |  | | 19 | | 5 | 1.00 |  |
| Ischemic | 414 | 104 | 0.59 (0.27-1.28) | 0.18 | | 240 | | 17 | 1.21 (0.34-4.34) | 0.77 | | 174 | | 87 | 0.53 (0.19-1.46) | 0.22 |
| Mixed | 17 | 2 | 1.26 (0.24-6.51) | 0.78 | | 9 | | 0 | - | - | | 8 | | 2 | 1.05 (0.17-6.60) | 0.96 |
| **Number of lesions:** |  |  |  |  | |  | |  |  |  | |  | |  |  |  |
| Focal | 363 | 66 | 1.00 |  | | 250 | | 18 | 1.00 |  | | 113 | | 48 | 1.00 |  |
| Multiple | 122 | 48 | 0.46 (0.30-0.71) | 0.00* | | 34 | | 2 | 1.22 (0.27-5.51) | 0.79 | | 88 | | 46 | 0.81 (0.50-1.33) | 0.41 |
| **Location of lesions:** |  |  |  |  | |  | |  |  |  | |  | |  |  |  |
| Unclear | 241 | 21 | 1.00 |  | | 230 | | 15 | 1.00 |  | | 11 | | 6 | 1.00 |  |
| Clear | 244 | 93 | 0.23 (0.14-0.38) | 0.00* | | 54 | | 5 | 0.70 (0.25-2.02) | 0.52 | | 190 | | 88 | 1.18 (0.42-3.29) | 0.76 |
| **Complications in acute phase:** | |  |  |  | |  | |  |  |  | |  | |  |  |  |
| Negative | 222 | 82 | 1.00 |  | | 134 | | 12 | 1.00 |  | | 88 | | 70 | 1.00 |  |
| Positive | 263 | 32 | 3.04 (1.94-4.74) | 0.00* | | 150 | | 8 | 1.68 (0.67-4.23) | 0.27 | | 113 | | 24 | 3.75 (2.18-6.43) | 0.00* |
| **3. Related Risk Factors** | |  |  |  | |  | |  |  |  | |  | |  |  |  |
| **Hypertension:** |  |  |  |  | |  | |  |  |  | |  | |  |  |  |
| Negative | 68 | 26 | 1.00 |  | | 32 | | 4 | 1.00 |  | | 36 | | 22 | 1.00 |  |
| Positive | 417 | 88 | 1.81 (1.09-3.01) | 0.02* | | 252 | | 16 | 1.97 (0.62-6.25) | 0.25 | | 165 | | 72 | 1.40 (0.77-2.55) | 0.27 |
| **Hyperlipidemia:** |  |  |  |  | |  | |  |  |  | |  | |  |  |  |
| Negative | 292 | 71 | 1.00 |  | | 166 | | 13 | 1.00 |  | | 126 | | 58 | 1.00 |  |
| Positive | 193 | 43 | 1.09 (0.72-1.66) | 0.68 | | 118 | | 7 | 1.32 (0.51-3.41) | 0.57 | | 75 | | 36 | 0.96 (0.58-1.59) | 0.87 |
| **Coronary heart disease:** | |  |  |  | |  | |  |  |  | |  | |  |  |  |
| Negative | 300 | 81 | 1.00 |  | | 176 | | 15 | 1.00 |  | | 124 | | 66 | 1.00 |  |
| Positive | 185 | 33 | 1.51 (0.97-2.36) | 0.07 | | 108 | | 5 | 1.84 (0.65-5.21) | 0.25 | | 77 | | 28 | 1.46 (0.87-2.48) | 0.16 |
| **Arrhythmia:** |  |  |  |  | |  | |  |  |  | |  | |  |  |  |
| Negative | 453 | 107 | 1.00 |  | | 263 | | 18 | 1.00 |  | | 190 | | 89 | 1.00 |  |
| Positive | 32 | 7 | 1.08 (0.46-2.51) | 0.86 | | 21 | | 2 | 0.72 (0.16-3.31) | 0.67 | | 11 | | 5 | 1.03 (0.35-3.06) | 0.96 |
| **Diabetes:** |  |  |  |  | |  | |  |  |  | |  | |  |  |  |
| Negative | 362 | 87 | 1.00 |  | | 208 | | 14 | 1.00 |  | | 154 | | 73 | 1.00 |  |
| Positive | 123 | 27 | 1.10 (0.68-1.77) | 0.71 | | 76 | | 6 | 0.85 (0.32-2.30) | 0.75 | | 47 | | 21 | 1.06 (0.59-1.90) | 0.84 |
| **Arteriosclerosis:** |  |  |  |  | |  | |  |  |  | |  | |  |  |  |
| Negative | 318 | 72 | 1.00 |  | | 211 | | 13 | 1.00 |  | | 107 | | 59 | 1.00 |  |
| Positive | 167 | 42 | 0.90 (0.59-1.38) | 0.63 | | 73 | | 7 | 0.64 (0.25-1.67) | 0.37 | | 94 | | 35 | 1.48 (0.90-2.45) | 0.13 |
| **Anemia:** |  |  |  |  | |  | |  |  |  | |  | |  |  |  |
| Negative | 477 | 111 | 1.00 |  | | 280 | | 18 | 1.00 |  | | 197 | | 93 | 1.00 |  |
| Positive | 8 | 3 | 0.62 (0.16-2.38) | 0.49 | | 4 | | 2 | 0.13 (0.02-0.75) | 0.02* | | 4 | | 1 | 1.89 (0.21-17.13) | 0.57 |
| **Smoking history:** |  |  |  |  | |  | |  |  |  | |  | |  |  |  |
| Never | 289 | 79 | 1.00 |  | | 142 | | 9 | 1.00 |  | | 147 | | 70 | 1.00 |  |
| Cessation | 99 | 19 | 1.42 (0.82-2.47) | 0.21 | | 73 | | 7 | 0.66 (0.24-1.85) | 0.43 | | 26 | | 12 | 0.76 (0.28-2.05) | 0.59 |
| Occasional | 17 | 1 | 4.65 (0.61-35.46) | 0.14 | | 9 | | 0 | - | - | | 8 | | 1 | 4.04 (0.48-34.17) | 0.20 |
| Frequent | 80 | 15 | 1.46 (0.80-2.67) | 0.22 | | 60 | | 4 | 0.95 (0.28-3.21) | 0.94 | | 20 | | 11 | 0.85 (0.32-2.20) | 0.73 |
| **Alcohol consumption:** |  |  |  |  | |  | |  |  |  | |  | |  |  |  |
| Never | 338 | 84 | 1.00 |  | | 180 | | 12 | 1.00 |  | | 158 | | 72 | 1.00 |  |
| Cessation | 75 | 10 | 1.86 (0.92-3.76) | 0.08 | | 54 | | 5 | 0.72 (0.24-2.13) | 0.55 | | 21 | | 5 | 2.51 (0.72-8.71) | 0.15 |
| Occasional | 41 | 18 | 0.57 (0.31-1.04) | 0.07 | | 24 | | 2 | 0.80 (0.17-3.79) | 0.78 | | 17 | | 16 | 0.52 (0.22-1.24) | 0.14 |
| Frequent | 31 | 2 | 3.85 (0.90-16.42) | 0.07 | | 26 | | 1 | 1.73 (0.22-13.89) | 0.60 | | 5 | | 1 | 2.89 (0.26-31.59) | 0.39 |
| **Diet type:** |  |  |  |  | |  | |  |  |  | |  | |  |  |  |
| [Vegetarian](javascript:void(0);) | 227 | 45 | 1.00 |  | | 160 | | 9 | 1.00 |  | | 67 | | 36 | 1.00 |  |
| Normal | 186 | 51 | 0.72 (0.46-1.13) | 0.15 | | 83 | | 8 | 0.58 (0.22-1.57) | 0.29 | | 103 | | 43 | 1.10 (0.61-1.98) | 0.76 |
| Meatier | 72 | 18 | 0.79 (0.43-1.46) | 0.45 | | 41 | | 3 | 0.77 (0.20-2.97) | 0.70 | | 31 | | 15 | 1.03 (0.44-2.40) | 0.95 |
| **Diet flavor:** |  |  |  |  | |  | |  |  |  | |  | |  |  |  |
| Light | 173 | 42 | 1.00 |  | | 117 | | 7 | 1.00 |  | | 56 | | 35 | 1.00 |  |
| Normal | 153 | 41 | 0.91 (0.56-1.47) | 0.69 | | 49 | | 3 | 0.98 (0.24-3.94) | 0.97 | | 104 | | 38 | 1.72 (0.93-3.17) | 0.08 |
| Salty | 159 | 31 | 1.25 (0.75-2.08) | 0.40 | | 118 | | 10 | 0.71 (0.26-1.92) | 0.50 | | 41 | | 21 | 1.36 (0.65-2.84) | 0.41 |
| **Exercise:** |  |  |  |  | |  | |  |  |  | |  | |  |  |  |
| Inactivity | 209 | 54 | 1.00 |  | | 80 | | 4 | 1.00 |  | | 129 | | 50 | 1.00 |  |
| Frequent | 66 | 29 | 0.59 (0.35-.1.00) | 0.05 | | 26 | | 0 | - | - | | 40 | | 29 | 0.59 (0.32-1.08) | 0.09 |
| Every day | 210 | 31 | 1.75 (1.08-2.83) | 0.02* | | 178 | | 16 | 0.56 (0.18-1.72) | 0.31 | | 32 | | 15 | 0.79 (0.38-1.68) | 0.54 |
| * Statistically significant differences (*p* < 0.05).  BMI, body mass index; CI, confidence interval; N-PCSI, non-post-stroke cognitive impairment; OR, odds ratio; PSCI, post-stroke cognitive impairment. | | | | | | | | | | | | | | | | |
