# Supplementary material for: Prevalence of Post-Stroke Cognitive Impairment in China: A Community-Based, Cross-Sectional Study
Source: PLoS One. 2015 Apr 13;10(4):e0122864. doi: 10.1371/journal.pone.0122864 (PMC4395303; doi:10.1371/journal.pone.0122864)
Supplement: S2 Table — (DOC) [file pone.0122864.s002.doc]

**Table S2**. Comparison of the difference between stroke survivors included and not in age and gender.

|  | **Stroke survivors included (N=599)** | **Stroke survivors not included (N=617)** | **x^2^/*t*** | ***P-*value** |
| --- | --- | --- | --- | --- |
| Age(Year) | 67.91±16.57 | 68.53±10.21 | -.917 | .359 |
| Gender (female) | 324 (54.09%) | 321 (52.03%) | 0.520 | .471 |
